# Integrins: roles in cancer development and as treatment targets

**DOI:** 10.1038/sj.bjc.6601576

**Published:** 2004-02-03

**Authors:** H Jin, J Varner

**Affiliations:** 1 John and Rebecca Moores Comprehensive Cancer Center, University of California, San Diego, 9500 Gilman Drive, La Jolla, CA 92093-0912, USA; 2 Department of Medicine, University of California, San Diego, 9500 Gilman Drive, La Jolla, CA 92093-0912, USA

**Keywords:** angiogenesis, metastasis, apoptosis, integrin *α*5*β*1, integrin *α*v*β*3

## Abstract

The integrin family of cell adhesion proteins promotes the attachment and migration of cells on the surrounding extracellular matrix (ECM). Through signals transduced upon integrin ligation by ECM proteins or immunoglobulin superfamily molecules, this family of proteins plays key roles in regulating tumour growth and metastasis as well as tumour angiogenesis. Several integrins play key roles in promoting tumour angiogenesis and tumour metastasis. Antagonists of several integrins (*α*5*β*1, *α*v*β*3 and *α*v*β*5) are now under evaluation in clinical trials to determine their potential as therapeutics for cancer and other diseases.

During the last 10 years, novel insights into the mechanisms that regulate cell survival as well as cell migration and invasion have led to the development of novel integrin-based therapeutics for the treatment of cancer. Several integrins play important roles in promoting cell proliferation, migration and survival *in vitro* and *in vivo*. Antagonists of these integrins suppress cell migration and invasion of primary and transformed cells and also induce apoptosis of primary cells. Integrin antagonists also block tumour angiogenesis and tumour metastasis. Currently, humanised antibody antagonists of integrins *α*5*β*1 and *α*v*β*3 as well as peptide inhibitors of integrins *α*v*β*3/*α*v*β*5 are under evaluation as angiogenesis-inhibiting therapeutics in cancer clinical trials.

## INTEGRINS REGULATE CELL SURVIVAL AND MIGRATION

**Figure 1:**
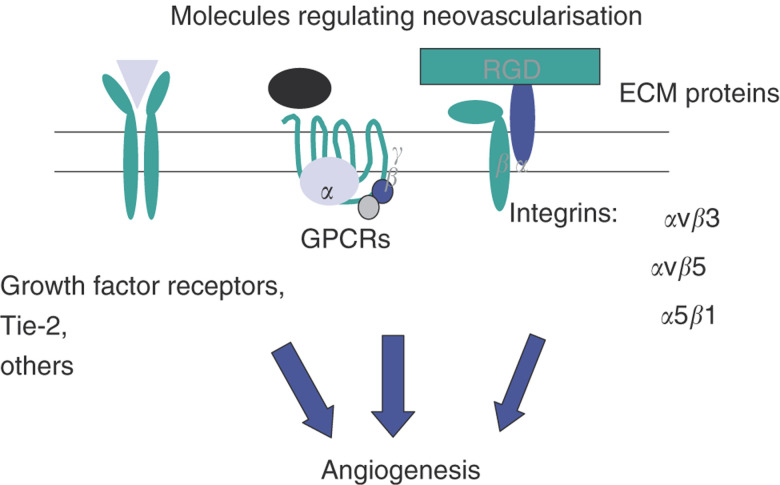
Molecules regulating angiogenesis. Growth factor receptors, other tyrosine kinase receptors such as Tie-2, G-protein-coupled receptors for angiogenesis modulating protein s such as interleukin-8 and parathyroid hormone-related peptide (Bakre *et al*, 2002), as well as integrins play key roles in the promotion of angiogenesis.

The invasion and survival of cells *in vivo* controls embryonic development, angiogenesis, tumour metastasis and other physiological processes (Aplin *et al*, 1998; Carmeliet and Jain, 2000; Hood and Cheresh, 2002). Cell surface receptors for the extracellular matrix (ECM), such as the integrins, play key roles in the regulation of normal and tumour cell migration and survival. The integrin family of cell adhesion proteins controls cell attachment to the ECM (Figure 1

). While some integrins selectively recognise primarily a single ECM protein ligand (e.g., *α*5*β*1 recognises primarily fibronectin), others can bind several ligands (e.g., integrin *α*v*β*3 binds vitronectin, fibronectin, fibrinogen, denatured or proteolysed collagen, and other matrix proteins). Several integrins recognise the tripeptide Arg–Gly–Asp (e.g., *α*v*β*3, *α*5*β*1, *α*IIb*β*3), whereas others recognise alternative short peptide sequences (e.g., integrin *α*4*β*1 recognises EILDV and REDV in alternatively spliced CS-1 fibronectin). Inhibitors of integrin function include function-blocking monoclonal antibodies, peptide antagonists and small molecule peptide mimetics matrix (reviewed in Hynes, 1992; Cheresh, 1993).

Although integrins mediate cellular adhesion to ECM proteins found in intercellular spaces and basement membranes, they also transduce intracellular signals that promote cell migration (reviewed in Aplin *et al*, 1998; Schwartz and Shattil, 2000) as well as cell survival (Meredith *et al*, 1993; Stromblad and Cheresh, 1996). However, unlike growth factor receptors, integrins have no intrinsic enzymatic activity but activate signalling pathways strictly by coclustering with kinases and adaptor proteins in focal adhesion complexes. The association of integrins with polyvalent or crosslinked ECM proteins clusters integrins and their associated cofactors, thus activating integrin-regulated signalling pathways. For example, integrin ligation suppresses apoptosis by activating suppressors of apoptosis (Pankov *et al*, 2003) and by inhibiting caspase activation (Stupack *et al*, 2001; Kim *et al*, 2002). Integrins also stimulate cell migration by activating Rho and Rac GTPases (Ren *et al*, 1999) and by anchoring actin filaments to the membrane. These adhesion proteins promote cell cycle entry by stimulating expression of cyclins (Assoian and Schwartz, 2001). Integrin ligation, therefore, supports signal transduction cascades that promote cell proliferation, cell survival and cell migration. In contrast, inhibition of cell integrin–ligand interaction inhibits cell migration (Kim *et al*, 2000a, 2000b; Bakre *et al*, 2002) and proliferation and induces apoptosis (Meredith *et al*, 1993; Boudreau *et al*, 1996; Stupack *et al*, 2001; Bakre *et al*, 2002; Kim *et al*, 2002).

## INTEGRIN ROLES IN CELL SURVIVAL

Studies from several groups showed that cell attachment is required for the survival of normal cells (Meredith *et al*, 1993; Stupack *et al*, 2001; Bakre *et al*, 2002). Complete loss of cell contact with the substratum (e.g., suspension culture) or adhesion to a nonspecific substratum such as poly-L-lysine induces apoptosis (‘anoikis’) of primary cells such as fibroblasts (Meredith *et al*, 1993), endothelial cells (Bakre *et al*, 2002; Kim *et al*, 2002) and epithelial cells (Frisch and Francis, 1994; Boudreau *et al*, 1996; Stupack *et al*, 2001). In contrast, loss of contact with the substratum does not necessarily kill tumour cells. Anchorage-independent tumour cells survive loss of contact with the substrate because they accumulate mutational changes in survival factors, such as upregulation of Bcl 2 expression and/or loss of p53 activity, which render the cells independent of integrin-mediated survival signals.

Recent studies have shown that cell death can also occur when a subset of integrins in a cell fail to bind their ECM ligands (Stupack *et al*, 2001; Kim *et al*, 2002). For example, expression of *α*v*β*3 or *α*5*β*1 can inhibit cell survival in cells attached to the matrix through other integrins (Stupack *et al*, 2001; Kim *et al*, 2002). The expression of *α*v*β*3 inhibits cell survival in cells attached to native collagen through integrin *α*2*β*1 (Stupack *et al*, 2001). As integrin *α*v*β*3 does not bind native collagen, these results indicate that the unligated integrin *α*v*β*3 induces cell death. In a similar manner, inhibition of integrin *α*5*β*1 activity with antibody antagonists induces apoptosis of endothelial cells that are attached to vitronectin through *α*v integrins (Kim *et al*, 2002). In addition, expression of dominant negative integrins (e.g., Tac-*β*3, the IL-2 receptor fused with the integrin beta 3 subunit cytoplasmic tail) also inhibits survival by impairing normal integrin-mediated survival signalling (Stupack *et al*, 2001). Integrin ligation suppresses caspase 8 activation, while unligated integrins facilitate caspase 8 activation in a stress response and death receptor independent manner (Stupack *et al*, 2001; Kim *et al*, 2002). Additional studies suggest that unligated integrins activate membrane-associated protein kinase A (PKA), which itself can activate caspase 8 in endothelial cells (Kim *et al*, 2002). Thus, in normal cells, some integrins regulate survival when ligated and induce apoptosis when unligated.

## INTEGRIN ROLES IN CELL MIGRATION

While integrin ligation by the ECM positively regulates migration, antagonising integrins inhibits cell migration. Although blocking integrin ligation can prevent cell attachment to the ECM and thus inhibit migration, recent studies show that antagonised integrins actively inhibit signal transduction leading to cell migration (Kim *et al*, 2000b). For example, the inhibition of integrin *α*5*β*1 negatively regulates fibroblast, endothelial cell and tumour cell migration even when other integrin receptors for provisional matrix proteins are ligated (Kim *et al*, 2000b). Antagonists of integrin *α*5*β*1 suppress cell migration on vitronectin, but not cell attachment to vitronectin, indicating that these antagonists affect the migration machinery rather than integrin receptors for vitronectin (Kim *et al*, 2000b). In fact, *α*5*β*1 antagonists activate PKA, which then inhibits cell migration by disrupting the formation of stress fibres (Kim *et al*, 2000b). Direct activation of PKA by forskolin or by overexpression of the catalytic, active subunit of PKA also inhibits cell migration (Bakre *et al*, 2002; Kim *et al*, 2000b). Thus, integrins regulate cell migration by making contact with the substratum and by promoting signal transduction cascades that support migration.

## INTEGRINS REGULATE ANGIOGENESIS

Angiogenesis is the process by which new blood vessels develop from pre-existing vessels. The growth of new blood vessels promotes embryonic development, wound healing and the female reproductive cycle, and also plays a key role in the pathological development of solid tumour cancers, haemangiomas, diabetic retinopathy, age-related macular degeneration, psoriasis, gingivitis, rheumatoid arthritis and possibly osteoarthritis and inflammatory bowel disease (reviewed in Carmeliet and Jain, 2000). New advances in understanding the mechanisms regulating angiogenesis, such as those that promote cell migration and invasion, are leading to the development of novel therapeutics for cancer.

**Figure 2:**
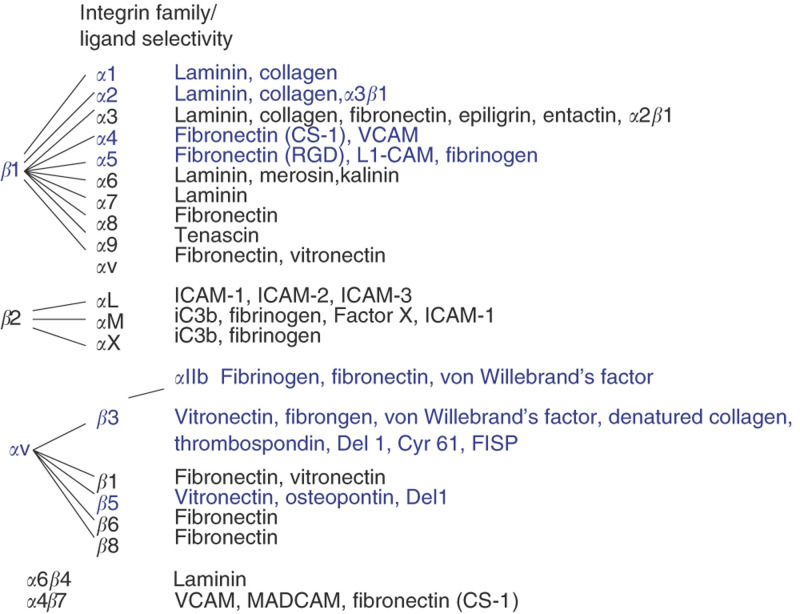
Integrin family. Integrin alpha beta heterodimers can be grouped into three subfamilies. Integrins *α*1*β*1, *α*2*β*1, *α*5*β*1, *α*4*β*1, *α*v*β*3 and *α*v*β*5 (highlighted in blue) have been shown to play important roles in regulating tumour angiogenesis.

Growth factors released by hypoxic tissues or pathological tissues such as tumours stimulate new blood vessel growth. New vessels grow by sprouting from pre-existing vessels (reviewed in Carmeliet and Jain, 2000) or by recruitment of bone marrow-derived endothelial progenitor cells (Asahara *et al*, 1997). While growth factors and their receptors play key roles in angiogenic sprouting, adhesion to the ECM also regulates angiogenesis (Figure 2

). Adhesion promotes endothelial cell survival (Kim *et al*, 2002; Stupack and Cheresh, 2002), as well as endothelial cell proliferation and motility (Kim *et al*, 2000a, 2000b) during new blood vessel growth. One ECM protein in particular, fibronectin, is associated with vascular proliferation (Kim *et al*, 2000a, 2000b); it is expressed in provisional vascular matrices and provides proliferative signals to vascular cells during wound healing, atherosclerosis and hypertension. Notably, fibronectin-null mice die early in development from a collection of defects, which include an improperly formed vasculature (George *et al*, 1993, 1997). Recent experimental studies showed that fibronectin regulates angiogenesis, as antibody inhibitors of fibronectin block angiogenesis (Kim *et al*, 2000a).

Studies in experimental angiogenesis models and in mutant mice indicate that several integrins play key roles in regulating angiogenesis. Embryonic deletion of integrin *α*5*β*1 induces early mesenchymal abnormalities, which include defects in the organisation of the emerging vasculature (Yang *et al*, 1993; Goh *et al*, 1997) and defects in the ability of endothelial cells to form vessel-like structures *ex vivo* (Taverna and Hynes, 2001; Francis *et al*, 2002). Similarly, loss of integrin *α*4*β*1 leads to aorta, heart and other vascular malformations (Yang *et al*, 1995). Deletion of the *α*v subunit causes 80% of embryos to die early in development from uncertain causes, while the few surviving embryos die a few hours after birth with significant defects in brain development, including failure of blood vessels to form properly (Bader *et al*, 1998). In contrast, individual loss of the *β*3or*β*5 subunit during embryogenesis does not cause noticeable defects in the formation of the cardiovascular system (Hodivala-Dilke *et al*, 1999; Huang *et al*, 2000). In fact, one study show that loss of the *β*3 or the *β*3 and *β*5 subunits promotes tumour angiogenesis (Reynolds *et al*, 2002). These studies led to the controversial conclusion that the *α*vβ3/*α*vβ5 integrins are not required for angiogenesis, but instead may suppress angiogenesis. As many studies have shown that *α*v*β*3 and *α*v*β*5 inhibitors block angiogenesis by inducing apoptosis in proliferating endothelial cells (Brooks *et al*, 1994b), it is possible that loss of the integrin-mediated death mechanism can lead to enhanced angiogenesis (Cheresh and Stupack, 2002). In fact, loss of either the *β*3 or *β*5 subunits does block angiogenesis induced by the angiomatrix protein Del-1 (Zhong *et al*, 2003). Thus, integrins appear to have diverse roles in the establishment of the cardiovascular system, with integrin *α*5*β*1 clearly playing a major role during development of the vascular system.

Studies in experimental models of angiogenesis also indicate that several integrins can play important roles in regulating angiogenesis in normal animals. The expression of both integrins *αvβ*3 (Brooks *et al*, 1994a) and *α*5*β*1 (Kim *et al*, 2000a) are significantly upregulated on endothelium during angiogenesis. The expression of integrins *α*v*β*3 and *α*5*β*1 partially controls angiogenesis; neither integrin is expressed by quiescent endothelium and both are expressed in response to angiogenic growth factors (Brooks *et al*, 1994a; Kim *et al*, 2000a, 2000b). Their expression is controlled by the transcription factor Hox D3 (Boudreau *et al*, 1997; Boudreau and Varner, 2003; Zhong *et al*, 2003). Hox D3 is a Homeobox gene expressed by ECs that may regulate an angiogenic switch. When expressed *in vivo*, Hox D3 promotes a haemangioma-like proliferation of blood vessels (Boudreau *et al*, 1997; Zhong *et al*, 2003); this transcription factor promotes the expression of integrin *α*v*β*3, *α*5*β*1 and uPA, molecules with established roles in angiogenesis. Thus, Hox D3 may provide a switch to activate a program of angiogenesis. Once integrins *α*5*β*1 and *α*v*β*3 are expressed, angiogenesis depends on each integrin as antagonists of each can block angiogenesis *in vivo* (Brooks *et al*, 1994a, 1994b; Kim *et al*, 2000a). Antibody and peptide antagonists of integrins *α*v*β*3 and *α*5*β*1 inhibit growth factor as well as tumour angiogenesis, tumour growth and tumour metastasis (Brooks *et al*, 1994a, 1994b; Carron *et al*, 1998; Kim *et al*, 2000a; Stoeltzing *et al*, 2003). These studies indicate that these integrins function in part by promoting survival in proliferating endothelial cells *in vivo* (Brooks *et al*, 1994b; Kim *et al*, 2002). Studies of the signals transduced when integrins are antagonised indicate that unligated integrins activate PKA, which then activates caspase 8 and induces apoptosis (Bakre *et al*, 2002; Kim *et al*, 2002).

In addition, other integrins have been shown to regulate angiogenesis. Integrin *α*v*β*5 promotes VEGF-, but not bFGF-, mediated angiogenesis (Friedlander *et al*, 1995). Integrin receptors for laminin and collagen also play roles in regulating blood vessel formation as antagonists of *α*2*β*1 and *α*1*β*1 suppressed VEGF-mediated angiogenesis (Senger *et al*, 1997). Thus, integrins play key roles in regulating tumour angiogenesis, and integrin antagonists hold promise as future therapeutics for cancer.

## INTEGRINS PLAY ROLES IN TUMOUR INVASION AND METASTASIS

Tumour metastasis promotes the spread of tumours to local and distant sites away from primary tumours. Metastasis is the leading cause of the morbidity and mortality associated with cancer. Tumour cells isolated from metastases are highly migratory and invasive. Therefore, understanding the mechanisms regulating cell migration may be helpful in developing new modes of therapy for metastatic cancer.

Increased levels of expression of integrins *α*v*β*3 is closely associated with increased cell invasion and metastasis (Felding-Habermann *et al*, 2002). Notably, integrin *α*v*β*3 is expressed on invasive melanoma but not benign nevi or normal melanocytes (Gehlsen *et al*, 1992). Additionally, increased *α*v*β*3 expression levels correlate with increased rates of melanoma metastases (Nip *et al*, 1992).

Integrin *α*6 expression is also significantly upregulated in numerous carcinomas, including head and neck cancers and breast cancer (Garzino-Demo *et al*, 1998; Mercurio *et al*, 2001; Ramos *et al*, 2002). Integrin *α*6*β*4 expression enhances tumour cell invasiveness and metastasis, particularly in breast carcinomas (Mercurio *et al*, 2001; Ramos *et al*, 2002). Thus, antagonists of these integrins may be useful to prevent the spread of tumour cells.

## INTEGRIN INHIBITORS AS THERAPEUTIC AGENTS FOR CANCER

Several integrin inhibitors are currently under investigation as therapeutics for cancer. Antibody and peptide inhibitors of integrins *α*v*β*3 and *α*v*β*5 (for review, see Kerr *et al*, 2002) and of *α*5*β*1 are currently in clinical trials for the inhibition of angiogenesis in cancer. A humanised anti-*α*v*β*3 antibody, Vitaxin, is currently in Phase II trials for cancer (Gutheil *et al*, 2000; Patel *et al*, 2001; Posey *et al*, 2001; Mikecz, 2000), while a humanised anti-*α*5*β*1 antibody is in Phase I trials for cancer (Varner, personal communication; www.pdl.com). A cyclic peptide inhibitor of integrin *α*v*β*3/*α*v*β*5, Cilengitide, is in Phase I/II trials for glioblastoma and other cancers (Burke *et al*, 2002; Eskens *et al*, 2003; Smith, 2003). Other promising integrin *α*5*β*1- and *α*v*β*3-blocking peptides with antitumour angiogenesis and tumour metastasis activities are currently in preclinical development (Carron *et al*, 1998; Reinmuth *et al*, 2003; Stoeltzing *et al*, 2003). As Avastin, the antibody inhibitor of VEGF, has recently shown promise as a therapeutic for colon cancer in Phase III clinical trials (Fernando and Hurwitz, 2003), these integrin-based antiangiogenesis therapeutics hold great promise as powerful therapeutics for the treatment of cancer.

## CONCLUSION

The studies reviewed here indicate that integrin promote cellular migration and survival in tumour and primary cells. Antagonists of integrins *α*v*β*3, *α*5*β*1, *α*v*β*5 and *α*6*β*4 show great promise as potential inhibitors of tumour growth and metastasis as well as tumour angiogenesis. Clinical trials are currently underway to evaluate inhibitors of integrin *α*v*β*3, *α*v*β*5 and *α*5*β*1 for their usefulness in the treatment of cancer.
